# Pulses, Healthy, and Sustainable Food Sources for Feeding the Planet

**DOI:** 10.3390/ijms18020255

**Published:** 2017-01-25

**Authors:** Marcello Iriti, Elena Maria Varoni

**Affiliations:** 1 Dipartimento di Scienze Agrarie e Ambientali, Università degli Studi di Milano, via G. Celoria 2, 20133 Milan, Italy; 2 Dipartimento di Scienze Biomediche, Chirurgiche ed Odontoiatriche, Università degli Studi di Milano, via Beldiletto 1/3, 20142 Milan, Italy; elena.varoni@unimi.it

Pulses, a subgroup of legumes, are plant foods from the Fabaceae (Leguminosae) family. According to FAO (Food and Agriculture Organization of the United Nations), pulses are annual leguminous crops, used for both food and feed, yielding from 1 to 12 grains or seeds of variable size, shape, and color within a pod. It was worth nothing that FAO does not consider pulses crops used as vegetables (e.g., green peas and green beans), for oil extraction (e.g., soybean and groundnut), or for sowing purposes (e.g., clover and alfalfa) (http://www.fao.org/es/faodef/fdef04e.htm). Following resolution 6/2013 of the 38th FAO Conference, the UN General Assembly, at its 68th session, declared 2016 as the International Year of Pulses.

Pulses are produced all over the world, with India representing the largest producer followed by the Russian Federation and Poland (Figure 1 and Figure 2). Worldwide, commonly eaten pulses include common beans (*Phaseolus vulgaris* L.), faba beans (*Vicia faba* L.), chickpeas (*Cicer arietinum* L.), peas (*Pisum sativum* L.), mung beans (*Vigna radiata* L.), cowpeas and black-eyed peas (*Vigna unguiculata* (L.) Walp.), and several varieties of lentils (*Lens culinaris* Medik.). Other less known species of pulses include lupines (e.g., *Lupinus albus* L. and *Lupinus mutabilis* Sweet) and bambara beans (*Vigna subterranea* L.). In various traditional cuisines, pulses are main components of typical dishes and recipes such as falafel (a patty made with fava beans) and hummus (a food dip made from chickpeas) in Egypt and Lebanon, respectively, githeri (a meal of maize and any type of beans mixed and boiled together) in Kenia, sopa de habichuelas negras (a black bean soup) in Dominican Republic, Dal (a soup with lentils, peas, or beans) in India, and farinata (a pancake with chickpea flour) in Italy, to cite a few (http://www.fao.org/in-action/inpho/resources/cookbook/en/).

Pulses are rich in macronutrients, i.e., carbohydrates (55%–65% of the total weight), mainly starches, and proteins (usually 21%–26%), including essential amino acids, and low in calories and fat (1%–4%) (Table 1) [1]. Pulses also contain significant amounts of micronutrients, namely minerals, water-soluble and lipid-soluble vitamins, and healthy lipids such as polyunsaturated fatty acids (Table 1).

In addition, pulses are good sources of bioactive components that are not considered as nutrients and typically occur in small quantities (when compared with macronutrients), but otherwise exert beneficial metabolic effects on the human body upon consumption in physiological conditions. These non-nutrient food constituents vary in concentration amongst different pulse species and varieties, and include non-digestible carbohydrates (soluble and insoluble dietary fibres, resistant starches and oligosaccharides) and bioactive phytochemicals, mainly polyphenols and phytosterols (Table 2).

In particular, pulse seed coats are rich in polyphenols, powerful antioxidants, while cotyledons contain phytosterols, effective cholesterol-lowering agents (Figure 3). Singh et al. have recently reviewed the health-promoting effects of pulses [2].

However, in many Western cultures, pulses are underestimated and considered “a poor man’s food” or “protein for the poor.” In truth, raw pulses also contain anti-nutrients, mainly phytates and tannins, which can reduce the intestinal absorption of metals, such as iron and zinc [3]. Furthermore, some non-digestible carbohydrates found in pulses can cause bloating and flatulence, and, not least, pulses require a much longer cooking time than vegetables. Fortunately, soaking dried pulses in water for 4–8 h as well as sprouting and fermentation reduce their anti-nutrient content, cooking time, and propensity to cause flatulence. Therefore, soaking ensures that pulses can be more easily digested, and their nutrients better absorbed by the gastrointestinal tract. Moreover, when pulses are combined with other foods, particularly grains, their nutritional value is further enhanced. In fact, the proteins of pulses are high in lysine and low in sulfur-containing amino acids, whereas the proteins of cereals are low in lysine but high in sulfur-containing amino acids [4]. Combining them provides a higher protein quality, as it occurs in many traditional dishes: feijoada (black beans and rice) in Brazil, mercimek köftesi (patties made from lentils and bulgur wheat) in Turkey, koshari (lentils and rice) in Egypt, waakye (beans and rice) in Ghana, pasta e fagioli (beans and pasta) in Italy, and kwati (a mixed soup of nine types of sprouted beans) in Nepal, to cite a few.

Besides their food value, pulses also play an important role in cropping systems. They do not require nitrogen fertilizers because of their nitrogen-fixing properties, thus increasing soil fertility. In addition, these plants are deep rooting and require less water than other crops to grow, which means pulses can tolerate detrimental environmental conditions such as drought and can grow in dry, arid lands where the majority of poor farmers reside and are unable to cultivate other crops. Therefore, these protein-rich plants can be cultivated in marginal areas and serve as a food source for most populations in developing countries, where meat, dairy, and fish are unavailable or too expensive.

In conclusion, pulses play a major role in addressing the future global food security and environmental challenges, and the International Year of Pulses will certainly raise the public awareness of the nutritional benefits of pulses as a relevant and indispensable component of a balanced and healthy diet.

## Figures and Tables

**Figure 1:**
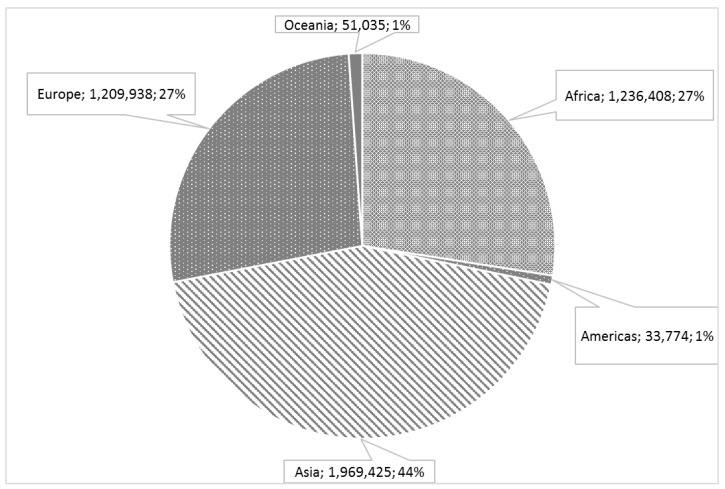
World pulse production (tonnes) by regions in 2014 (http://faostat.fao.org).

**Figure 2:**
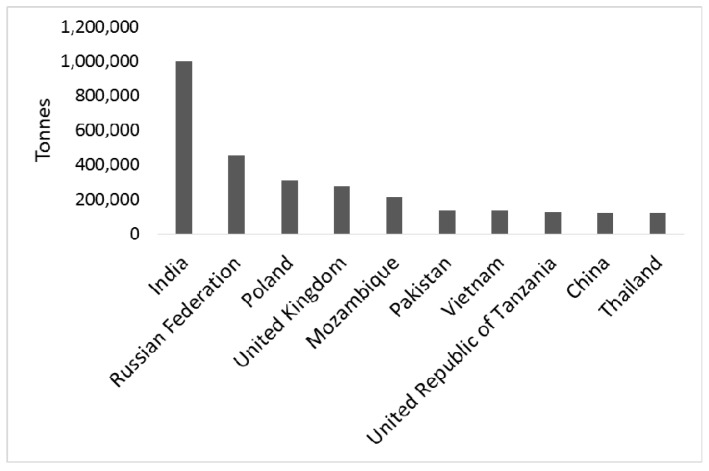
Top 10 pulse producing countries in 2014 (http://faostat.fao.org).

**Figure 3:**
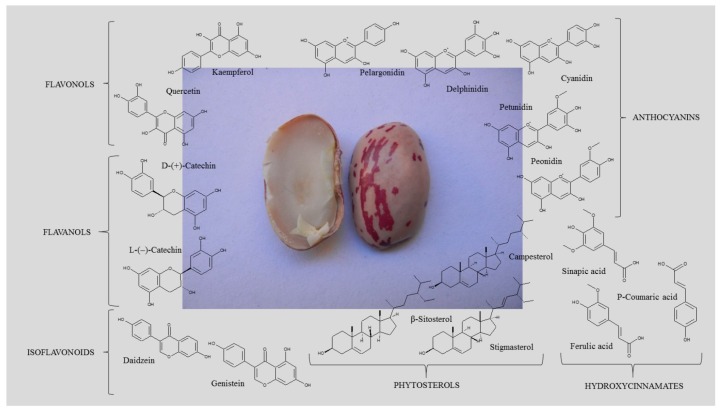
Occurrence of the main phenolic compounds and phytosterols in common bean (*Phaseolus vulgaris* L.) seed tissues; phenolic compounds include hydroxycinnamates (ferulic acid, p-coumaric acid, and sinapic acid), anthocyanins (glycosides of cyanidin, delphinidin, pelargonidin, peonidin and petunidin), flavonols (glycosides of kaempferol and quercetin), flavanol units of proanthocyanidins (catechins), and isoflavonoids (daidzein and genistein); main phytosterols include β-sitosterol, stigmasterol, and campesterol.

**Table 1:** Nutritional composition of common bean (*Phaseolus vulgaris* L.), cranberry, mature seed, raw *.

Constituents	Value per 100 g
Water	12.39 g
Energy	335 kcal
Fiber (total)	24.7 g
Ash	3.31 g
**Nutrients**	**Value per 100 g**
Proteins	23.03 g
Lipids (total)	1.23 g
Carbohydrates	60.05 g
**Minerals**	**Value per 100 g**
Calcium (Ca)	127 mg
Iron (Fe)	5 mg
Magnesium (Mg)	156 mg
Phosphorous (P)	372 mg
Potassium (K)	1332 mg
Sodium (Na)	6 mg
Zinc (Z)	3.63 mg
Copper (Cu)	0.794 mg
Manganese (Mn)	0.920 mg
Selenium (Se)	12.7 µg
**Vitamins**	**Value per 100 g**
Vitamin B1 (thiamin)	0.747 mg
Vitamin B2 (riboflavin)	0.213 mg
Vitamin B3 (niacin)	1.455 mg
Vitamin B5 (pantothenic acid)	0.748 mg
Vitamin B6 (piridoxine)	0.309 mg
Folate (total)	604 µg
Vitamin A	2 IU
**Lipids**	**Value per 100 g**
Fatty acids (total saturated)	0.316 g
14:0	0.001 g
16:0	0.296 g
18:0	0.019 g
Fatty acids (total monounsaturated)	0.106 g
18:1 (undifferentiated)	0.106 g
Fatty acids (total polyunsaturated)	0.527 g
18:2 (undifferentiated)	0.287 g
18:3 (undifferentiated)	0.240 g
**Amino acids**	**Value per 100 g**
Tryptophan	0.273 g
Threonine	0.969 g
Isoleucine	1.017 g
Leucine	1.838 g
Lysine	1.580 g
Methionine	0.346 g
Cystine	0.251 g
Phenylalanine	1.245 g
Tyrosine	0.648 g
Valine	1.205 g
Arginine	1.426 g
Histidine	0.641 g
Alanine	0.965 g
Aspartic acid	2.785 g
Glutamic acid	3.511 g
Glycine	0.899 g
Proline	0.976 g
Serine	1.253 g

* Source: U.S. Department of Agriculture Database Nutrient Database for Standard Reference (http://nal.usda.gov, retrieved on 4 January 2017).

**Table 2:** Phenolic composition of common bean (*Phaseolus vulgaris* L.), black, whole, raw on fresh weight (FW) basis *.

Phenolic acids	Compound	Mean Content (mg 100 g^−1^ FW)
Hydroxycinnamic acids		
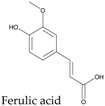	Caffeic acid	0.73
	Ferulic acid	20.63
	*p*-Coumaric acid	9.47
	Sinapic acid	7.17
	
**Flavonoids**		**Mean Content (mg 100 g^−1^ FW)**
Anthocyanins	Cyanidin	1.63
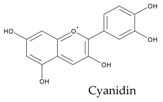	Cyanidin 3,5-*O*-diglucoside	1.98
	Cyanidin 3-*O*-glucoside	3.99
	Delphinidin 3-*O*-feruloyl-glucoside	1.10
	Delphinidin 3-*O*-glucoside	14.50
	Malvidin 3-*O*-glucoside	0.60
	Pelargonidin	0.95
	Pelargonidin 3,5-*O*-diglucoside	1.54
	Pelargonidin 3-*O*-glucoside	12.60
	Peonidin	1.36
	Petunidin 3-*O*-glucoside	0.80
**Flavonols**	Kaempferol	1.80
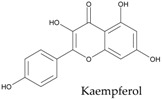	Kaempferol (after hydrolysis)	0.88
	Kaempferol 3-*O*-acetyl-glucoside	3.40
	Kaempferol 3-*O*-glucoside	6.60
	Quercetin (after hydrolysis)	0.98
**Isoflavonoids**		
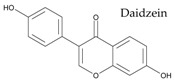	Daidzein	0.80
	Genistein	0.60
	
	
**Proanthocyanidins**		**Mean Content (mg 100 g^−1^ FW)**
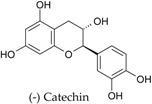		
	Flavanol monomers	2.90
	Flavanol dimers	5.20
	
	
**Total polyphenols (Folin assay)**	**1390.75 mg 100 g^−1^ FW**	

* Source: Phenol Explorer—Database on polyphenol content in foods (http://phenol-explorer.eu, retrieved on 4 January 2015).

## References

[1] Tharanathan R.N., Mahadevamma S. (2003). Grain Legumes—A Boon to Human Nutrition. Trends Food Sci. Technol..

[2] Singh B., Singh J.P., Shevkani K., Singh N., Kaur A. (2016). Bioactive constituents in pulses and their health benefits. J. Food Sci. Technol..

[3] Haileslassie H.A., Henry C.J., Tyler R.T. (2016). Impact of household food processing strategies on antinutrient (phytate, tannin and polyphenol) contents of chickpeas (*Cicer arietinum* L.) and beans (*Phaseolus vulgaris* L.): A review. Int. J. Food Sci. Technol..

[4] Rebello C.J., Greenway F.L., Finley J.W. (2014). Whole grains and pulses: A comparison of the nutritional and health benefits. J. Agric. Food Chem..

